# Lipid analysis of meat from Bactrian camel (*Camelus bacterianus*), beef, and tails of fat-tailed sheep using UPLC-Q-TOF/MS based lipidomics

**DOI:** 10.3389/fnut.2023.1053116

**Published:** 2023-03-02

**Authors:** Qingqing Li, Li Yang, Rongrong Li, Gangliang Chen, Jing Dong, Linying Wu, Yinghua Fu, Jie Yang

**Affiliations:** ^1^College of Life Science and Technology, Xinjiang University, Ürümqi, China; ^2^Xinjiang Camel Industry Engineering Technology Research Center, Ürümqi, China

**Keywords:** camel meat, hump, beef, fatty-tails, fatty acid, lipidomics, lipid metabolism

## Abstract

**Introduction:**

As a source of low-cost and high-quality meat for human beings, the consumption of camel meat was increasing, and beef has similar texture and nutritional characteristics with camel meat. Camel hump and fatty-tails are important parts of fat storage for camels and fat-tailed lambs, respectively, which were to adapt and endure harsh environments. Considering their similar physiological functions, their fat composition might be similar. Lipidomics is a system-level analysis of lipids method, which play an important role in the determination and quantification of individual lipid molecular specie, food adulteration and labeling.

**Methods:**

A GC/MS was used to analyze fatty acids composition of Xinjiang Bactrian camel meat, hump, beef, and fatty-tails. UPLC-Q-TOF/MS based on lipidomics approach was used to analyze lipid composition, characterize and examine the lipid differences in Xinjiang Bactrian camel meat, hump, beef, and fatty-tails.

**Results and discussion:**

The major fatty acids of the four samples were C16:0, C18:0, and C18:1*cis*, and camel meat had a significant low SFA content and high MUFA content. A total of 342 lipid species were detected, 192, 64, and 79 distinguishing lipids were found in the groups camel hump compared to camel meat, camel meat compared to beef, and camel hump compared to fatty-tails, respectively. Lipid metabolisms of ether lipid, glycerophospholipid, glycerolipid, and sphingolipid were the most influential pathways revealed by KEGG analysis. The results contributed to enrich the lipid information of Bactrian camel meat, and indicated that UPLC-Q-TOF/MS based on lipidomics was an alternative method to distinguish meat samples.

## 1. Introduction

Dietary lipids comprise glycerol and long-chain fatty acids, which can provide essential nutrients for maintaining human health and contribute to various intra and extracellular signaling pathways (1). Dietary lipids are also involved in many pathological processes, such as microbial imbalance, metabolic complications and neuropathological processes (2). Meat is rich in lipids, and the different content and type in meat have been linked to dietary health concerns, plus contribute as important flavor components (3). Demand for meat continues to grow due to human population growth. Camel, an under-used and accessible animal food source, could be a suitable option to fulfill the demand gap in the meat market (4), and camel carcass can provide a large amount of edible meat for humans. Recently, the consumption of camel meat is increasing due to its low cost and high-quality meat, especially in some African and Asian countries where camel meat is more accessible than other meat (5). FAOSTAT data shows that the annual world camel meat production is approximately 600,000 tons (6).

The camelids are divided into dromedaries and Bactrian camels, dromedaries mainly living in hot arid countries from Africa, Middle East and South Asia, while Bactrian camels (*Camelus bacterianus*) are generally found in Central Asia and China (7). High-quality camel food products such as milk and meat are important for people in semi-arid and arid areas due to their unique physiological characteristics (8). The nutritional composition of camel meat has been reported as high in moisture, protein content but low in fat cholesterol and ash content. Camel meat was rich in EAA such as leucine (LEU), Lysine (LYS), valine (VAL) which were dietary essential amino acids; the content of Fe, Mn and Na, which are necessary to the human diet and healthy food, were high level in camel meat; and camel meat contained significantly higher levels of vitamins B, E, as compared with beef, chicken, and mutton (9). Camel meat also has a high indispensable to dispensable amino acids (IEAA: EAA), reasonable polyunsaturated: saturated fatty acids (PUFA: SFA, P: S) ratios, and high percentage of C18:2 (*cis* 9 and *trans* 11) (10). Raiymbeka et al. reported that the mean value of essential amino-acid index of dromedary meat and Bactrian meat were 216.9 and 191.6, respectively, which was high compared to other red meats, and both meats were rich in methionine and leucine (7). Based on these benefits of camel meat, coupled with the pursuit of a low-fat and healthy diet, camel meat may be healthier's choice for human consumption. Likewise, beef is also characterized by high protein and low-fat content, and the taste and texture of meat from young camels (below three-year-old) is comparable to beef (11). The hump is another important part of the Bactrian camel, as it is a food source due to its high fat content. The hump of the dromedary camel accounts for about 8.6% of the carcass weight (8). The camel's hump has specialized immune and endocrine functions and osmoregulatory roles, which are important for their survival in the harsh desert environment (12). Moreover, Iranian fat-tailed lambs store large quantities of fat in their fatty-tails when food is plentiful; these fat stores can be decomposed into energy when food is scarce to adapt and endure harsh environments and winters (13). Perhaps the fat composition of fatty-tails is like camel humps, considering their similar physiological role and accumulation on the respective body parts.

Lipidomics, a system-level analysis of lipids on a large scale, would apply to detect food adulteration and labeling, and help to qualify, and quantify individual lipid molecular specie (14). To an obtain comprehensive lipid profile, various analytical methods based on lipomics were used applied to quantify trace lipid molecules in foods. UPLC-ESI-Q-TOF-MS was used to investigate the lipid metabolites after being fed different PUFA diets of Landrace pig muscle, and lipid composition (15). Four, three and eight lipids were determined as potential chemical markers in pork cuts, and black pig varieties were analyzed using UPLC-ESI-MS/MS, respectively (16). Many lipids, such as glycerophospholipids, were found to be the marker candidates between chicken meat and abdominal fat from different sources with an LC-MS-based lipidomics approach (14). At present, lipidomics has been widely used in various research fields, such as dairy product adulteration, disease diagnosis, mechanism of food lipid oxidation mechanism (17–20).

In this study, the meat lipids from Xinjiang Bactrian camels, beef, and fatty-tails were analyzed using UPLC-Q-TOF/MS based on lipidomics to establish a characterization of the lipid profiles. The lipid datasets of groups, camel meat compared with camel hump, camel meat compared with beef, and camel hump compared with fatty-tails, were analyzed to identify the differential lipids and related lipid metabolisms. The study will provide lipid information from Bactrian camel meat and camel hump, give lipid species clue to discriminate camel meat and beef, camel hump and fatty-tails.

## 2. Material and methods

### 2.1. Chemicals

LC-MS grade methanol, methyl tert-butyl ether (MTBE) and water were purchased from CNW Technologies (Dusseldorf, Germany). LC-MS grade ammonium acetate, ammonium hydroxide, acetonitrile (ACN), dichloromethane (DCM) and isopropanol (IPA) were supplied by Merck (Darmstadt, Germany). The standards d7-LPC (18:1), d7-PE (15:0/18:1) and d7-TG (15:0/18:1/15:0) were supplied by Avanti Inc. (Alabama, USA).

### 2.2. Preparation of meat samples

The *longissimus lumborum* (LL) and hump of Bactrian camels were supplied by Xinjiang Wangyuan Biological Technology Group Co., Ltd. (Xinjiang, China); the male camel was about 4 years old. The LL of beef (*Bos Taurus*) and tails of fat-tailed sheep (*Ovis Aries*) were obtained from a same distributor. The LL were taken from the 13th/14th rib immediately after slaughter, quick-frozen, stored at −80 °C for further analysis.

### 2.3. Histology

Meat samples (2 × 1 × 1 cm) were trimmed and were fixed using 10% formalin. The samples were dehydrated with gradient ethanol, serially hyalinized in xylene: ethanol (v/v, 1:1) and xylene, and the tissue mass was imbedded in paraffin wax. After, the samples were sliced to 5-μm thickness by a microtome, and fixed between a slide and cover slip. Sections were dewaxed and rehydrated using xylene, gradient ethanol, and distilled water. After each section was stained with hematoxylin and eosin, the samples mounted using neutral resins after being dehydrated and hyalinized. Finally, images were taken using an ETHOS1 microscope (LabTechq, Beijing, China) at a 100× magnification.

### 2.4. Fatty acids analysis

The Folch method was used to extract the fat (21). The fat transesterification/methylation procedures followed: 40 mg of fat were weighted in tubes, 240 μL of 10% BF_3_-methanol were added and heated at 100°C water for 90 min. After cooling to laboratory temperature, the samples added 480 μL of n-hexane and 1.5 mL of deionized water. Shaken the samples and centrifuged at 2,000 rpm for 5 min, collected the organic phase (upper layer). The upper solution was repeatedly washed (deionized water) and centrifuging (2,000 rpm, 5 min) three time. After that, 0.2 g of anhydrous sodium sulfate was added to the samples and held at 4 °C for 30 min. Finally, the samples were centrifuged (2,000 rpm, 5 min) and collected upper solution.

Fatty acid analysis was performed by a GC/MS (70090B/5977A, Agilent Technologies, USA) with a FID detector, using a DB – 5MS capillary column (30 m long, film 0.25 μm thickness and 0.25 mm internal diameter). The GC parameters: helium flow rate, 1.0 mL/min; vaporization chamber temperature, 250 °C; injection volume, 1 μL; split ratio, 30:1. The oven temperature started at 80 °C for 5 min, then which was increased to 260 °C at 5 °C/min and held for 15 min. The MS parameters: temperature of the inlet, 290 °C; ionization mode, EI; ion source temperature, 200 °C; electron energies, 70 ev; scan of collecting in m/z range 3–550 amu.

### 2.5. Analysis of lipidomics

#### 2.5.1. Lipid extraction procedure

The lipid fraction was extracted with MTBE. The muscle samples (camel meat and beef) were minced, and 10 mg was precisely weighed, and was spiked with 1,600 μL of MS-grade water in a glass bottle with a screw cap. After, 200 μL of the homogenization sample was diluted with 200 μL of water. Likewise, 3 mg of the adipose samples (hump and fatty-tails) were weighed and spiked with 400 μL of MS-grade water. 960 μL of MTBE: methanol (v/v, 5:1), which comprised 9 μL of 10 ppm d7-PE, 9 μL of 10 ppm d7-LPC, and 9 μL of 100 ppm d7-TG, was added to the bottles. After the samples vortex-mixed for 60 s and ultra-sonicated for 10 min, the mixture was frozen centrifuged at 3,000 rpm for 15 min. Collected the upper solution (organic phase), and the lower layer was re-extracted with 500 μL of MTBE for extraction twice more. The collected organic phases were combined and evaporated under nitrogen. Lipid extractions were re-dissolved by 100 μL of DCM: methanol (v/v, 1:1) and 60 μL of the solution was removed to analysis. Five replications of each sample were performed.

#### 2.5.2. UPLC-Q-TOF/MS conditions

Lipids analysis was performed with 1290 UPLC (Agilent, USA) equipped with Triple TOF 6600 (Q-TOF, AB Sciex, USA), using a Phenomen Kinetex C18 100A Column (100 × 2.1 mm, 1.7 μm). The mobile phase A was comprised 10 mM Ammonium acetate, 40% H_2_O and 60% ACN, and the mobile phase B was comprised 10 mM Ammonium acetate, 10% CAN and 90% IPA. An elution gradient started with 40% B for 12 min; subsequently changed to 100% B within 1.5 min; at 13.7 min, changed to 40% B again. Run duration was 18 min with 300 μL/min. The injection volumes were 1 μL (positive mode) and 3 μL (negative mode). The Analyst TF 1.7 (AB Sciex, USA) constantly assessed the comprehensive scan survey MS data as it collected and touched off the acquisition of MS/MS spectra depending on preselected criteria. The program of ESI source: ion source gas 1 and 2 were 60, curtain gas was 30; the source temperature was held at 550°C, ion spray voltage floating was 5,500 and −4,500 V.

### 2.6. Statistics analysis

The original data format was converted to mzXML using the ProteoWizard version 3.0.6150 (California, USA). The mzXML document imported into Lipid Analyzer for analysis. An MS/MS spectral library were used for lipid identification. Variance (ANOVA), student's t-test, principal component analysis (PCA), and orthogonal projections to latent structures-discriminate analysis (OPLS-DA) were performed for data analysis. *p* value, variable importance in projection (VIP) value and fold change (FC) value were applied to differentiate the lipid composition between samples. The lipid species with VIP > 1 and *p* < 0.05 were statistically significant.

## 3. Results

### 3.1. Tissue sections of meat

**Figure 1:**
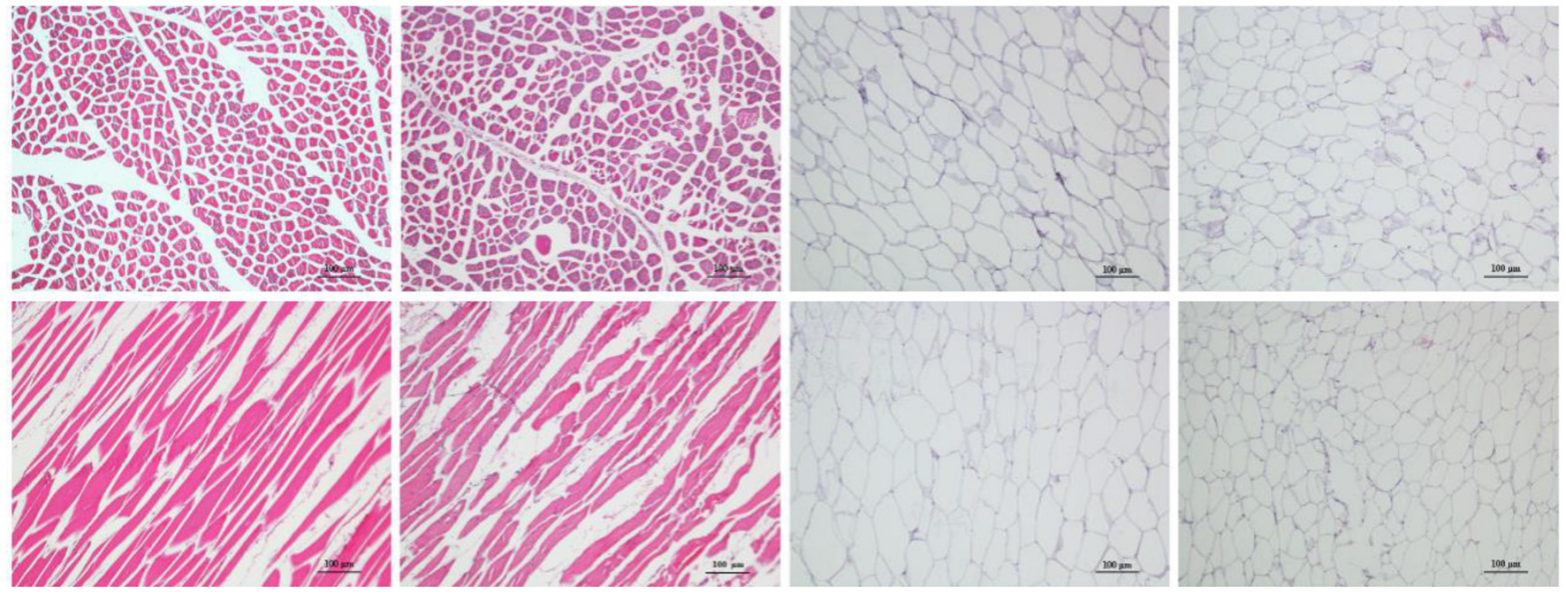
Representative sections from four meat samples by hematoxylin and eosin (H&E) staining (×100). From **left to right:** *Longissimus lumborum* of camel and beef, hump, and fatty-tails. **Top:** cross-sections; **bottom:** longitudinal sections. The scale bar represents 100 μm.

The histological sections of LL of meat from camel and beef as well as adipose tissue from the hump and fatty-tails were shown in Figure 1, displaying the distribution variation of fat. Visually, the muscle fiber structure was unbroken with shape of cable in muscle tissue from camel meat and beef, and comparing with beef, the camel muscle had a bright red color. The sizes of the camel muscle fibers were much smaller than the beef's, further, the extracellular spaces were smaller in the camel muscle. Moreover, unilocular adipocytes comprised most of the adipose tissue mass in hump and fatty-tails, and the cell size of the hump was larger than that of fatty-tails.

### 3.2. Analysis of fatty acids

The fatty acid compositions of camel meat, beef, hump and fatty-tails were shown in Table 1. The content of fatty acids was different, however, the composition of the major fatty acids, consistent in all meat, were C16:0, C18:0, and C18:1*cis*. The camel meat had a significantly lower content of SFAs than that of camel hump, beef, and fatty-tails (31.82 *vs*. 48.92, 37.69, 39.96%, respectively), short-chain SFAs (C11:0, C12:0, and C13:0) were only found in the fatty-tails (C11:0 was 0.61%, C12:0 was 0.09%) and hump (C13:0 was 0.20%), and most long-chain SFAs, such as C21:0, C22:0, C23:0, C24:0, were found in the hump with 0.10, 0.28, 0.06, 0.10%, respectively. The monounsaturated fatty acids (MUFAs) from camel meat, beef, camel hump and fatty-tails were 53.92, 43.61, 43.48, 49.97%, respectively. Oleic acid (C18:1 n-9 *cis*) was the most abundant MUFA, the second was elaidic acid (C18:1 n-9 *trans*), and camel meat (41.02%) had a higher content of oleic acid than beef (35.78%). Linoleic acid (LA, C18:2 *cis*), conjugated linoleic acid (CLA, C18:2), and arachidonic acid (ARA, C20: 4) were the main PUFA in all four samples. Camel meat and beef were rich in LA (4.83, 5.38%, respectively), ARA (1.31, 2.61%, respectively), and eicosapentaenoic acid (EPA, C20:5; 0.51, 1.49%, respectively), and CLA was mainly distributed in the camel meat (2.33%), hump (2.07%), and fatty-tails (2.22%). Moreover, there were more PUFAs in the camel meat than hump, such as CLA (2.33 *vs*. 2.07%), ALA (0.25 *vs*. 0.19%), C24:4 n-6 (0.12 *vs*. 0.08%). The ratios of n-6: n-3 PUFAs of camel meat, hump, beef, and fatty-tails were 6.98, 10.38, 3.29, and 13.87, respectively. The ratio of polyunsaturated: saturated fatty acids (PUFA: SFA, P: S) was similarly in camel meat and beef (0.31 *vs*. 0.28), hump and fatty-tails (0.11 *vs*. 0.12). As expected, the camel hump and fatty-tails had the lowest P: S ratio due to their higher content of SFA.

**Table 1:** Fatty acid composition of camel meat, camel hump, beef, and fatty-tails.

**Fatty acids**	**Camel meat**	**Camel hump**	**Beef**	**Fatty-tails**
**∑SFA**	31.82%	48.92%	37.69%	39.96%
C11:0	-	-	-	0.61%
C12:0	-	-	-	0.09%
C13:0	-	0.20%	-	-
C14:0	2.30%	2.95%	1.01%	2.73%
C15:0	0.24%	0.71%	0.26%	0.57%
C16:0	15.55%	18.76%	15.35%	17.98%
C17:0	0.57%	1.41%	1.07%	0.38%
C18:0	12.80%	23.23%	19.40%	17.18%
C19:0	0.13%	0.38%	0.16%	0.30%
C20:0	0.23%	0.72%	0.28%	0.11%
C21:0	-	0.10%	-	-
C22:0	-	0.28%	0.07%	-
C23:0	-	0.06%	-	-
C24:0	-	0.10%	0.09%	-
**∑MUFA**	53.92%	43.61%	43.48%	49.76%
C14:1	0.08%	0.06%	0.13%	
C16:1 n-9 *trans*	0.25%	0.42%	0.18%	0.06%
C16:1 n-9 *cis*	2.56%	2.29%	1.44%	2.24%
C17:1 n-9 *cis*	0.59%	0.93%	0.76%	1.32%
C18:1 n-9 *cis*	41.02%	34.91%	35.78%	39.96%
C18:1 n-9 *trans*	8.84%	3.78%	4.27%	5.98%
C19:1	0.54%	0.37%	0.26%	-
C20:1 n9	-	0.82%	0.65%	0.21%
C22:1 n9	0.04%	0.04%	-	-
**∑PUFA**	9.82%	5.15%	10.67%	4.96%
C18:2 n−6 (LA)	4.83%	2.30%	5.38%	2.21%
C18:2 n−6 (CLA)	2.33%	2.07%	-	2.22%
C18:3 n−3 (ALA)	0.25%	0.19%	-	-
C20:3 n−3	0.47%	0.27%	0.68%	0.25%
C20:4 n−6 (ARA)	1.31%	0.24%	2.61%	0.20%
C20:5 n−3 (EPA)	0.51%	-	1.49%	0.08%
C22:6 n−3 (DPA)	-	-	0.32%	-
C24:4 n−6	0.12%	0.08%	0.19%	-
**∑n−3**	1.23%	0.45%	2.49%	0.33%
**∑n−6**	8.59%	4.70%	8.18%	4.63%
**n−6:n−3**	6.98	10.38	3.29	13.87
**PUFA:SFA**	0.31	0.11	0.28	0.12

### 3.3. Lipid profiling

**Figure 2:**
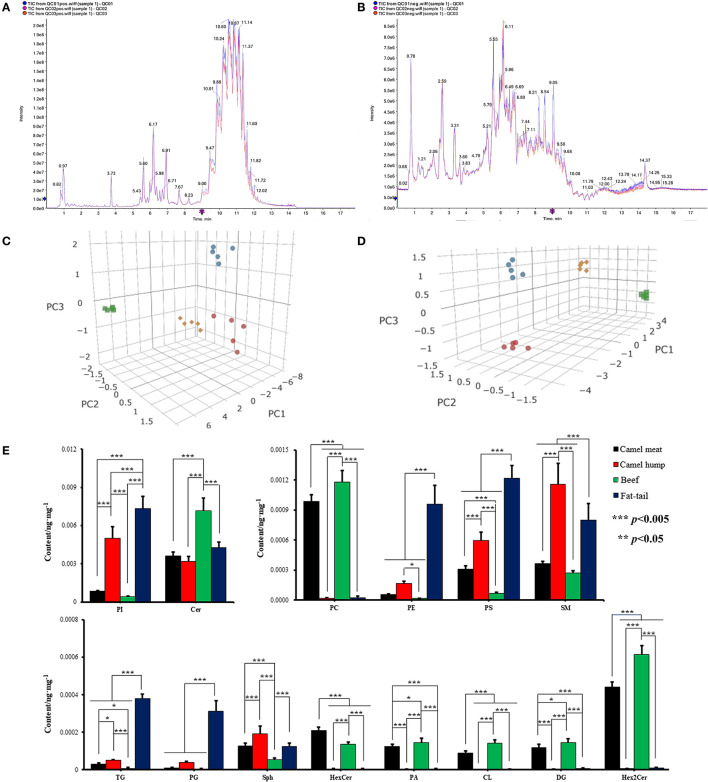
Representative TICs ESI positive **(A)** and ESI negative **(B)** ionization mode of meat. 3D PCA score plots of lipidomics dataset from camel meat (blue circle), camel hump (green square), beef (red circles) and fatty-tails (yellow rhombus) in ESI positive **(C)** and negative **(D)** ionization mode (*n* = 5). **(E)** The content of major lipids in different types of meat. PI, phosphatidylinositol; Cer, ceramide; PC, phosphatidylcholine; PE, phosphotidylethanolamine; PS, phosphatidylserine; SM, sphingomyelin; TG, triglyceride; PG, phosphatidylglycerol; Sph, sphinganine; HexCer, hexosylceramide; PA, phosphoric acid; CL, cephalin; DG, diglyceride; Hex2Cer, dihexosylceramide.

Representative TICs of camel meat, beef, camel hump and fatty-tails extracts were shown in Figures 2A, B. The signals of these extracts in positive ionization mode were mainly attributed to triglyceride (TG), phosphatidylcholine (PC), sphingomyelin (SM), ceramide (Cer), diglyceride (DG), and sphinganine (Sph). In the negative ionization mode, signals were mainly attributed to phosphatidylethanolamine (PE), phosphatidylinositol (PI), phosphatidylserine (PS), hexosylceramide (HexCer), dihexosylceramide. (Hex2Cer), phosphatidylcholine (PC), cephalin (CL), phosphatidylglycerol (PG), and phosphoric acid (PA). 342 lipid species were identified, and the total lipid molecules species and quantitative results were summarized in Supplementary material S1. Those 342 matches were assigned into 14 subclasses, comprising 104 PC, 76 PE, 7 PS, 11 PI, 5 CL, 8 PG, 3 PA, 9 Sph, 13 Cer, 1 HexCer, 6 Hex2Cer, 23 SM, 12 DG, and 64 TG (Supplementary Figure S1). An unsupervised PCA was performed for comparison of lipid differences between the camel meat, hump, beef, and fatty-tails. A complete separation between groups was revealed by the PCA score plots (Figures 2C, D), indicating that there were significantly different in lipids of camel meat, beef, hump and fatty-tails.

The mass percentages of the 14 lipid classes were calculated and comparison of major lipid subclass contents in camel meat, beef, camel hump and fatty-tails were shown in Figure 2E. The contents of Hex2Cer, DG, PC, HexCer, CL, and PA in the camel meat (0.44, 0.12, 0.99, 0.21, 0.090 ×10^−3^ ng/mg, respectively) and beef (0.61, 0.14, 1.2, 0.14, 0.14 ×10^−3^ ng/mg, respectively), were significantly higher than the camel hump (0.68, 0.23, 2.0, 0.49, 0.14 ×10^−5^ ng/mg, respectively) and fatty-tails (0.81, 0.54, 2.4, 0.49, 0.19 ×10^−5^ ng/mg, respectively) (*p* < 0.05). The contents of PI, SM, TG, and PS in muscle from camel meat (0.86, 0.37, 0.030, 0.31 ×10^−3^ ng/mg, respectively) and beef (0.41, 0.27, 0.0075, 0.069 ×10^−3^ ng/mg, respectively) were significantly lower than adipose tissue from hump (5.0, 1.2, 0.049, 0.60 ×10^−3^ ng/mg, respectively) and fatty-tails (7.3, 0.80, 0.38, 1.2 ×10^−3^ ng/mg, respectively). The content of HexCer (0.21 *vs*. 0.0050, 0.14, 0.0050 ×10^−3^ ng/mg) in the camel meat was significantly high (*p* < 0.005) comparing with the other samples, SM (1.5 ×10^−3^ ng/mg) and Sph (0.19 ×10^−3^ ng/mg) were more abundant in the hump, Cer, PC, PA, CL, DG, and Hex2Cer were higher in beef with 7.2, 1.2, 0.15, 0.14, 0.14, and 0.61 ×10^−3^ ng/mg, respectively (*p* < 0.05), and the contents of PI, PE, PS, TG, and PG in the fatty-tails were higher than the other samples with 7.3, 0.96, 1.2, 0.38, 0.31 ×10^−3^ ng/mg, respectively (*p* < 0.05).

### 3.4. Comparison of lipid profiles between the camel meat and camel hump

**Figure 3:**
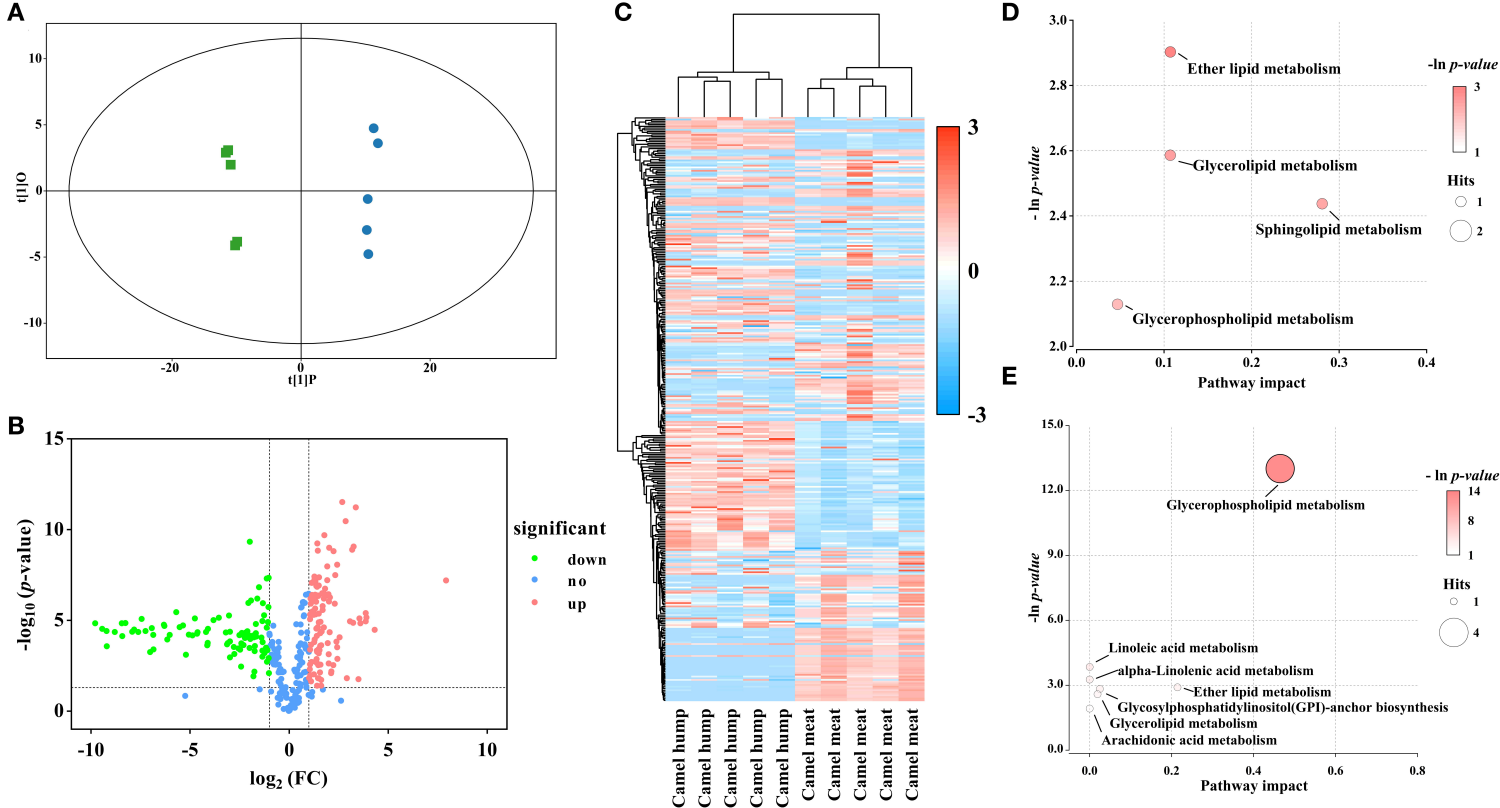
Statistical analyses of lipids in camel hump *vs*. camel meat. **(A)** OPLS-DA scores plot. Green squares represent camel hump, blue circles represent camel meat (R^2^Y= 0.996, Q^2^ = 0.991). **(B)** Volcano plot using log_2_ FC as the x-axis, -log_10_ (*p* < 0.05) as the y-axis, FC values were the ratios of lipid content in camel hump to that in camel meat. **(C)** Heat map analysis and hierarchical clustering of differential abundance lipids between camel hump and camel meat. To build the heat map, normalized abundances were used; the red color represents high abundance, whereas the blue color represents low abundance. Bubble plots of KEGG pathway analysis in positive **(D)** and negative **(E)** ionization mode, and the supplementary data showed in Supplementary material S2.

In the study, OPLS-DA scores were applied to elucidate intragroup separation and variables responsible for classification, A distinction was observed in the score scatter plot of OPLS-DA model for the group of camel hump compared with camel meat (Figure 3A). In addition, a volcano plot and hierarchical clustering were constructed, which showed the lipid composition of the camel hump and camel meat were significantly different (Figures 3B, C). A total of 236 lipids with differential abundance (VIP > 1 and *p* < 0.05). Among them, 192 species were found to have an FC < 0.5 or FC > 2, with 99 lipids were high in camel hump and 93 lipids were high in camel meat, which were potential markers for distinguishing between camel hump and camel meat groups (Supplementary material S1). To determine the possible pathways contributing to differentially expressed lipids, KEGG analysis was applied to identify metabolic pathways and their networks. A total of 8 lipid metabolisms were enriched (Supplementary material S2). The result of metabolic pathway analysis was shown in bubble plots (Figures 3D, E). The metabolic pathways of glycerophospholipid metabolism, sphingolipid metabolism, ether lipid metabolism, and glycerolipid metabolism have the most impact values (0.4649, 0.2807, 0.2143, and 0.1070, respectively) and might play a key role in developing lipids.

### 3.5. Comparison of lipid profiles between camel meat and beef

**Figure 4:**
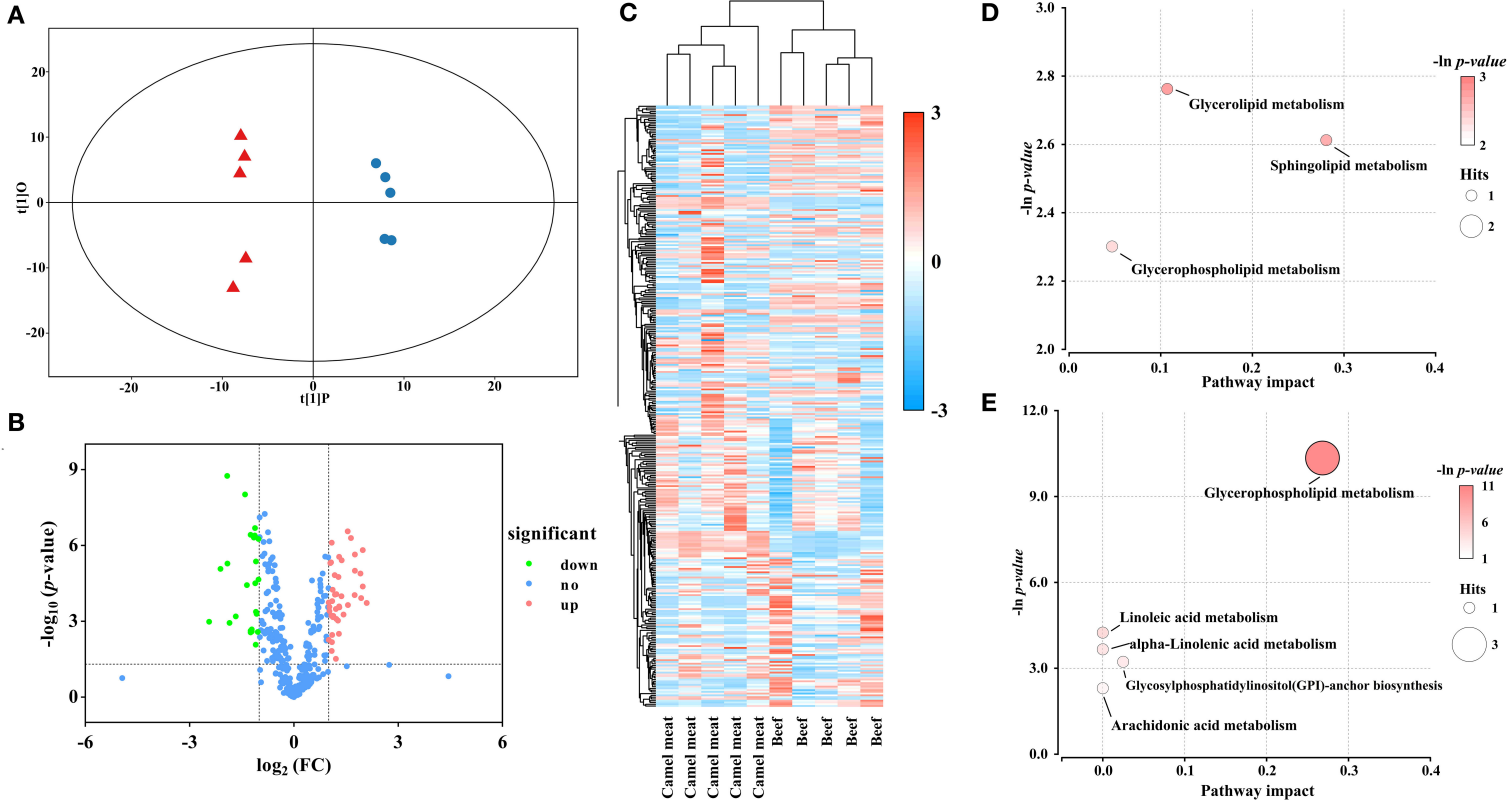
Statistical analyses of lipids in camel meat *vs*. beef. **(A)** OPLS-DA scores plot. Blue circles represent camel meat, red triangles represent beef (R^2^Y = 0.995, Q^2^ = 0.983). **(B)** Volcano plot using log_2_ FC as the x-axis, -log_10_ (*p* < 0.05) as the y-axis, FC values were the ratios of lipid content in camel meat to that in beef. **(C)** Heat map and hierarchical clustering of differential abundance lipids between camel meat and beef. To build the heat map, normalized abundances were used; the red color represents high abundance, whereas the blue color represents low abundance. Bubble plots of KEGG pathway analysis in positive **(D)** and negative **(E)** ionization mode, and the supplementary data showed in Supplementary material S2.

There was good visual separation between camel meat and beef group in OPLS-DA score plot (Figure 4A). Furthermore, the volcano plot presented a differential compound between camel meat and beef (Figure 4B). To identify the potential biomarkers from the differential metabolites, we clustered the differential metabolites using heat maps and hierarchical clustering (Figure 4C). The results showed that PLs, DGs, and TGs differed in camel meat and beef. Based on the OPLS-DA model and multivariate statistics (VIP > 1 and *p* < 0.05, FC < 0.5 or FC > 2), 64 individual lipid species were screened as potential molecules for distinction to the camel meat and beef (Supplementary material S1). 41 lipids were more abundant in camel meat than those in beef. Different expressed lipids of camel meat and beef were mainly related to seven lipid pathways (Supplementary material S2). Among the identified metabolic pathways, glycerolipid metabolism, sphingolipid metabolism, and glycerophospholipid metabolism were observed to be the most important, their impact values were 0.1070, 0.2807 and 0.2682, respectively (Figures 4D, E).

### 3.6. Comparison of lipid profiles between camel hump and fatty-tails

**Figure 5:**
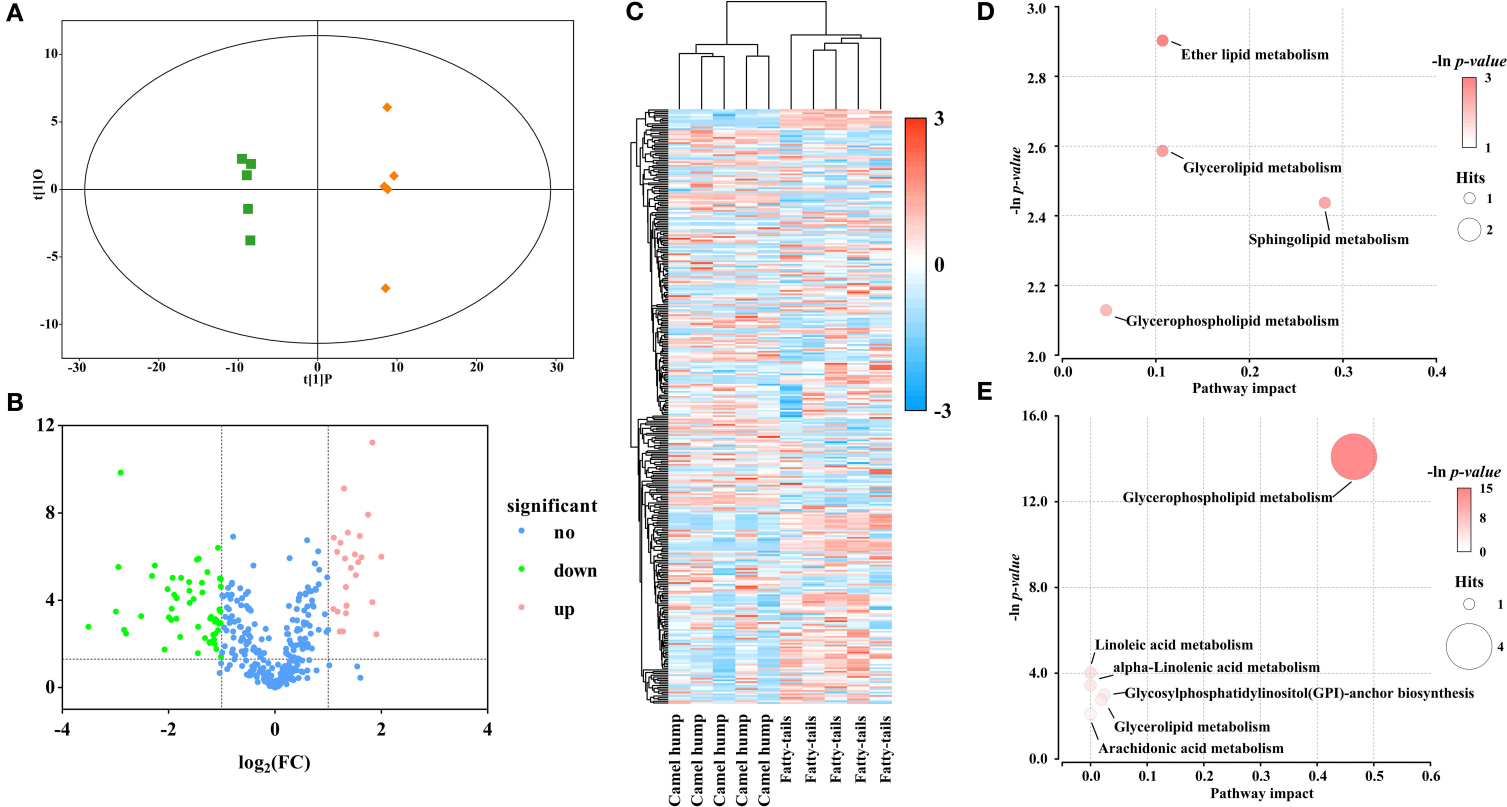
Statistical analyses of lipids in camel hump *vs*. fatty-tails. **(A)** OPLS-DA scores plot. Green squares represent camel hump, yellow rhombus represent fatty-tails (R^2^Y = 0.995, Q^2^ = 0.983). **(B)** Volcano plot using log_2_ FC as the x-axis, -log_10_ (*p* < 0.05) as the y-axis; FC values were the ratios of lipid content in camel hump to that in fatty-tails. **(C)** Heat map and hierarchical clustering of differential abundance lipids between camel hump and fatty-tails. To build the heat map, normalized abundances were used; the red color represents high abundance, whereas the blue color represents low abundance. Bubble plots of KEGG pathway analysis in positive **(D)** and negative **(E)** ionization mode, and the supplementary data showed in Supplementary material S2.

The camel hump and fatty-tails were also investigated to identify potential lipid markers. The result of OPLS-DA demonstrated that camel hump and fatty-tails samples were separated from each other, and a permutation test indicated that the model had robustness and reliability (Figure 5A). The volcano plot and heat map (Figures 5B, C) on the different metabolites presented the clustering of lipids with differential abundance between the camel hump and fatty-tails. A total of 79 lipid molecules (VIP > 1 and *p* < 0.05; FC < 0.5, FC > 2) were selected as chemical descriptors, 54 lipids were more abundant in camel hump compared with fatty-tails (Supplementary material S1). The result of pathway analysis showed that there were constructed eight metabolic pathways in the group (Supplementary material S2). Among the identified metabolic pathways, lipid metabolisms of ether lipid, glycerolipid, sphingolipid, and glycerophospholipid had the greatest impact on differential lipids, their values of impact were 0.1071, 0.1070, 0.2807, and 0.4649, respectively (Figures 5D, E).

## 4. Discussion

There was a difference in color, structure, and IMF content between the LL of camel and beef. Factors such as breed, diet, production systems, and genetics may influence display color traits variability and IMF deposition of muscle (22). Likewise, fat deposition of the adipose tissue in domestic animals was affected by the same factors, and was also influenced by resistin and leptin (23).

In this study, fatty acid contents and species were different in camel meat, beef, hump and fatty-tails. These differences are in agreement with the literatures (10). C16:0, C18:0, and C18:1*cis* were the major fatty acids in all samples. Compared with dromedary camel muscle, Xinjiang two-hump camels have higher content of C18:1 *cis* (41.02 *vs*. 25.9%), lower content of C16:0 (15.55 *vs*. 22.40%), and C18:0 (12.8 *vs*. 16.9%) (10). Furthermore, palmitic acid, stearic acid, and oleic acid were shown as the predominant mixtures of fatty acids in camel humps (24). Kadim et al. reported the proportions of C18:1 *cis* (33.5%) and total MUFAs (41.4%) in dromedary camel *longissimus thoracis*, which were lower than LL of camel meat from Xinjiang, the difference may be attributed to the camel species and cuts (25). Effectiveness of a MUFA-enriched diet to improve insulin sensitivity and associated cardiometabolic risk, as well as improve blood lipids and systemic inflammatory responses and endothelial dysfunction (26). The ratio of n-6: n-3 PUFAs was closely related to human health, including multiple biological processes, metabolic homeostasis, and diseases (27). Among the samples, the ratio of n-6: n-3 PUFAs in beef was within the recommended values of British Department of Health, which should not exceed 4.0 (28), in this respect, beef had better nutritional value than camel meat. The P:S is approximately 0.31 in camel meat, it was similar to the value (around 0.3) in dromedary camel indicated by Abdelhadi et al. (8). From a consumer-health viewpoint, nutritional advice for this ratio is 0.4 or higher (29). In the study, the ratio of P:S in beef (0.28) was slightly lower than camel meat, the value of beef was similar with *Limousin bulls* (0.29) (30), but camel meat was inconsistent with Maqsood et al. (4).

PI was the most abundant lipid class in the camel hump (5.0 ×10^−3^ ng/mg) and fatty-tails (7.3 ×10^−3^ ng/mg), the content of n-3 PUFAs-PI was high, and PI (18:0/20:4) was the major species. It had been reported that PI is the primary source of C20:4 n-6, and required for biosynthesis of eicosanoids (31). Beef has the highest content of Cer, which was associated with regulating cellular proliferation and apoptosis, and can be an important mediator of cancer-promoting effects of chronic alcohol consumption (32). PC was the main lipid differential species in camel meat and beef, accounting for about 25% of the total lipid differential species. PC is an important source of choline sphingomyelin and plasmalogen, and the supplement of choline in the form of phosphatidylcholine could reverse fatty livers (33).

To further explore the generation of differential lipid molecules, we used the KEGG pathway analysis to identify eight lipid metabolic pathways. Lipid metabolisms of ether lipid, glycerolipid, sphingolipid, and glycerophospholipid were the most influential pathways for three groups. LysoPC (O-18:0) was mainly enriched in ether lipid metabolism. Ether lipids were an abundant subclass of glycerophospholipid present in membranes, and were a fundamental building block of glycosylphosphatidylinositol (GPI) anchors (34). TG was a primary participant of glyceride metabolism, and serving as the primary form of energy storage in mammalian cells, synthesis and lipolysis of TG is associated with disease, for example, obesity, hypertension, and lipodystrophy (35). In the study, TGs in camel meat were significantly lower than in camel hump. TG was the major lipid class in adipose tissue (>90%), whereas there was a considerable proportion of PL in the muscle tissue (36). Here, the TGs with long-chain and medium-chain FAs were high in camel hump and fatty-tails, respectively. This was probably related to TGs containing long-chain and/or medium-chain fatty acids, and different metabolic processes to synthesize and store TGs (37). Besides, cold stimulates could also cause a highly selective increase in the abundance of long-chain/ultra-long-chain/odd-chain acyls TG (38).

We observed that Cer (d18:1/18:0) was more abundant in camel meat compared with camel hump, however, Cer (d18:1/18:0) and Cer (d18:1/24:1) were lower in camel meat than in beef. Cer intermediates in sphingolipid metabolism, as lipid second messengers, it played an important role in pathophysiological mechanisms, and the plasma concentrations of Cer increased after diet (39). However, recent research has indicated that the higher Cer (d18:1/18:0) and Cer (d18:1/24:1) concentrations in plasma were related with the development of adverse cardiovascular events (40). Differential metabolites of PLs, such as PC, PE, and PS, were mainly regulated by glycerophospholipid metabolism, and the majority were PC and PE. Dietary PL could improve antioxidant capacity, promote lipid utilization and reduce liver lipid accumulation (41). In the study, PUFAs had side chains coupled with mostly PL, such as PC (14:0/18:3, 18:2; 15:0/18:3, 18:2), PC (P-18:0/18:2, 18:3, 18:4, 20:5, 22:6; P-20:0/18:2, 18:4, 20:4, 22:6), PE (20:5; P-16:0/20:4, P-18:0/22:6), and PE (18:0/18:2; 18:1/18:2; 18:2/0:0, 18:2, 3:0). In ruminants, PUFA was preferentially deposited in phospholipids, their major role in metabolism and physiology helped to elucidate the potential beneficial effects of meat (42).

## 5. Conclusion

In this study, comparative lipidomics analysis of camel meat, camel hump, beef, and sheep fatty-tails were performed. There was a difference in fatty acid composition and camel meat had a significant low SFA content and high MUFA content. The potential lipid markers for distinguishing meat from different species were identified. PS (18:0/20:3), PI (16:0/20:4), PE (12:0/17:0) and DG (16:1/16:1), PC (P-16:0/17:2), TG (17:2/17:2/17:2) and TG (18:0/18:0/22:5), Hex2Cer (d14:1/16:0), TG (17:0/18:1/18:2) were the lipid components with the largest fold differences between camel meat and camel hump, camel meat and beef, camel hump to that in fatty-tails, respectively. UPLC-Q-TOF-MS based on lipidomics could be a feasible strategy to distinguish the meat samples. The results of pathway analysis suggested that ether lipid metabolism, glycerophospholipid metabolism, glycerolipid metabolism, and sphingolipid metabolism might be the most impactful to develop lipid profile in camel meat, hump, beef, and sheep fatty-tails. This study contributed to enrich lipids information of Bactrian camels, and provided a groundwork to discriminate camel meat, camel hump, beef, and fatty-tails. In the future, further investigation is required with a larger number of camel meat samples collected from different areas and breeds for an extended characterization of lipid profiles, meanwhile, comparison with other livestock meat is needed for exploring differences in lipids.

## Data availability statement

The original contributions presented in the study are included in the article/Supplementary material, further inquiries can be directed to the corresponding authors.

## Author contributions

QL: writing—original draft and writing—review and editing. LY: writing—original draft. RL: writing—review and editing. GC, JD, and LW: resources and funding acquisition. YF: conceptualization and writing—review and editing. JY: conceptualization, project administration, and funding acquisition. All authors contributed to the article and approved the submitted version.

## References

[1] Honda T Kabashima K. Current understanding of the role of dietary lipids in the pathophysiology of psoriasis. J Dermatol Sci. (2019) 94:314–20. 10.1016/j.jdermsci.2019.05.003 31133503

[2] Custers Emma EM Kiliaan Amanda J. Dietary lipids from body to brain. Prog Lipid Res. (2022) 85:101144. 10.1016/j.plipres.2021.101144 34915080

[3] Trivedi DK Hollywood KA Rattray NJW Ward H Trivedi DK Greenwood J. Meat, the metabolites: An integrated metabolite profiling and lipidomics approach for the detection of the adulteration of beef with pork. Analyst. (2016) 141:2155–64. 10.1039/c6an00108d 26911805 PMC4819684

[4] Maqsood S Abushelaibi A Manheem K Kadim IT. Characterisation of the lipid and protein fraction of fresh camel meat and the associated changes during refrigerated storage. J Food Compost Anal. (2015) 41:212–20. 10.1016/j.jfca.2014.12.027

[5] Abdel-Naeem HHS Mohamed HMH. Improving the physico-chemical and sensory characteristics of camel meat burger patties using ginger extract and papain. Meat Sci. (2016) 118:52–60. 10.1016/j.meatsci.2016.03.021 27045253

[6] FAOSTAT. Food and Agriculture Data. (2022). Available online at: https://www.fao.org/faostat/zh/#data/QCL (accessed October 18, 2022).

[7] Raiymbek G Kadim I Konuspayeva G Mahgoub O Serikbayeva A Faye B. Discriminant amino-acid components of Bactrian (*Camelus bactrianus*) and Dromedary (*Camelus dromedarius*) meat. J Food Compost Anal. (2015) 41:194–200. 10.1016/j.jfca.2015.02.006

[8] Kadim IT Mahgoub O Purchas RW A. review of the growth, and of the carcass and meat quality characteristics of the one-humped camel (*Camelus dromedaries*). Meat Sci. (2008) 80:555–69. 10.1016/j.meatsci.2008.02.010 22063567

[9] Hamed Hammad Mohammed H Jin G Ma M Khalifa I Shukat R Elkhedir AE. Comparative characterization of proximate nutritional compositions, microbial quality and safety of camel meat in relation to mutton, beef, and chicken. LWT. (2020) 118:108714. 10.1016/j.lwt.2019.108714

[10] Abdelhadi OMA Babiker SA Bauchart D Listrat A Rémond D Hocquette JF. Effect of gender on quality and nutritive value of dromedary camel (Camelus dromedarius) longissimus lumborum muscle. J Saudi Soc Agric Sci. (2017) 16:242–9. 10.1016/j.jssas.2015.08.003

[11] Dawood AA. Physical and sensory characteristics of Najdi-camel meat. Meat Sci. (1995) 39:59–69. 10.1016/0309-1740(95)80007-7 22059763

[12] Guo F Si R He J Yuan L Hai L Ming L. Comprehensive transcriptome analysis of adipose tissue in the Bactrian camel reveals fore hump has more specific physiological functions in immune and endocrine systems. Livest Sci. (2019) 228:195–200. 10.1016/j.livsci.2019.09.003

[13] Alizadeh A Zare Shahneh A Yousefi AR Hadinezhad Omran M Campbell AW. Determining the effect of the fat-tail and carcass weight on meat fatty acid composition of Iranian lambs. Small Ruminant Res. (2013) 115:34–9. 10.1016/j.smallrumres.2013.06.004

[14] Li J Li Z Ran J Yang C Lin Z Liu Y. LC/MS-based lipidomics to characterize breed-specific and tissue-specific lipid composition of chicken meat and abdominal fat. LWT. (2022) 163:113611. 10.1016/j.lwt.2022.113611

[15] Dannenberger D Nuernberg G Nuernberg K Will K Schauer N Schmicke M. Effects of diets supplemented with n−3 or n−6 PUFA on pig muscle lipid metabolites measured by non-targeted LC–MS lipidomic profiling. J Food Compost Anal. (2017) 56:47–54. 10.1016/j.jfca.2016.11.015

[16] Mi S Shang K Li X Zhang C-H Liu J-Q Huang D-Q. Characterization and discrimination of selected China's domestic pork using an LC-MS-based lipidomics approach. Food Control. (2019) 100:305–14. 10.1016/j.foodcont.2019.02.001

[17] Jia W Di C Zhang R Shi L. Application of liquid chromatography mass spectrometry-based lipidomics to dairy products research: an emerging modulator of gut microbiota and human metabolic disease risk. Food Res Int. (2022) 157:111206. 10.1016/j.foodres.2022.111206 35761528

[18] B A Waseem M Kashif M Srinivasan H. Lipidomics: an excellent tool for chronic disease detection. Curr Res Transl Med. (2022) 70:103346. 10.1016/j.retram.2022.103346 35487168

[19] Murphy RC Merrill AH. “Lipidomics.” Reference Module in Life Sciences. Amsterdam, Netherlands: Elsevier (2022).

[20] Zhao X Cheng X Zang M Wang L Li X Yue Y. Insights into the characteristics and molecular transformation of lipids in Litopenaeus vannamei during drying from combined lipidomics. J Food Compost Anal. (2022) 114:104809. 10.1016/j.jfca.2022.104809

[21] Folch J Lees M Sloane Stanley GH. A simple method for the isolation and purification of total lipides from animal tissues. J Biol Chem. (1957) 226:497–509. 10.3989/scimar.2005.69n187 13428781

[22] Mancini RA Hunt MC. Current research in meat color. Meat Sci. (2005) 71:100–21. 10.1016/j.meatsci.2005.03.003 22064056

[23] Lonergan SM Topel DG Marple DN. Fat and fat cells in domestic animals. In: The Science of Animal Growth and Meat Technology. (2019) p. 51–69. 10.1016/b978-0-12-815277-5.00005-6

[24] Kadim IT Mahgoub O Al-Maqbaly RS Annamalai K Al-Ajmi DS. Effects of age on fatty acid composition of the hump and abdomen depot fats of the Arabian camel (*Camelus dromedarius*). Meat Sci. (2002) 62:245–51. 10.1016/S0309-1740(01)00254-6 22061418

[25] Kadim IT Al-Ani MR Al-Maqbaly RS Mansour MH Mahgoub O Johnson EH. Proximate, amino acid, fatty acid and mineral composition of raw and cooked camel (Camelus dromedarius) meat. Br Food J. (2011) 113:482–93. 10.1108/00070701111123961

[26] Gillingham LG Harris-Janz S Jones PJH. Dietary monounsaturated fatty acids are protective against metabolic syndrome and cardiovascular disease risk factors. Lipids. (2011) 46:209–28. 10.1007/s11745-010-3524-y 21308420

[27] Alshatwi AA Subash-Babu P. Effects of increasing ratios of dietary omega-6/omega-3 fatty acids on human monocyte immunomodulation linked with atherosclerosis. J Funct Foods. (2018) 41:258–67. 10.1016/j.jff.2017.12.020

[28] Crawford T. UK nutritional aspects of cardiovascular disease. J Clin Pathol. (1996) 19:1–186.

[29] De la Fuente J Díaz MT Álvarez I Oliver MA Font i Furnols M Sañudo C. Fatty acid and vitamin E composition of intramuscular fat in cattle reared in different production systems. Meat Sci. (2009) 82:331–7. 10.1016/j.meatsci.2009.02.002 20416720

[30] Cuvelier C Clinquart A Hocquette JF Cabaraux JF Dufrasne I Istasse L. Comparison of composition and quality traits of meat from young finishing bulls from Belgian Blue, Limousin and Aberdeen Angus breeds. Meat Sci. (2006) 74:522–31. 10.1016/j.meatsci.2006.04.032 22063057

[31] Gardocki ME Jani N Lopes JM. Phosphatidylinositol biosynthesis: biochemistry and regulation. Biochim Biophys Acta Mol Cell Biol Lipids. (2005) 1735:89–100. 10.1016/j.bbalip.2005.05.006 15967713

[32] Barron KA Jeffries KA Krupenko NI. Sphingolipids and the link between alcohol and cancer. Chem Biol Interact. (2020) 322:109058. 10.1016/j.cbi.2020.109058 32171848 PMC8371698

[33] Takama K Suzuki T Yoshida K Arai H Mitsui T. Phosphatidylcholine levels and their fatty acid compositions in teleost tissues and squid muscle. Comp Biochem Physiol B Biochem Mol Biol. (1999) 124:109–16. 10.1016/S0305-0491(99)00115-7

[34] Jiménez-Rojo N Leonetti M Zoni V Colom A Feng S Iyengar N. Conserved function of ether lipids and sphingolipids in the early secretory pathway. Curr Biol. (2019) 30:3775–87.e7. 10.1101/2019.12.19.881094 32857977

[35] Ahmadian M Duncan RE Jaworski K Sarkadi-Nagy E Sul HS. Triacylglycerol metabolism in adipose tissue. Future Lipidol. (2007) 2:229–37. 10.2217/17460875.2.2.229 19194515 PMC2633634

[36] Wood JD Enser M Fisher AV Nute GR Sheard PR Richardson RI. Fat deposition, fatty acid composition and meat quality: a review. Meat Sci. (2008) 78:343–58. 10.1016/j.meatsci.2007.07.019 22062452

[37] Suzuki S Ishikawa S Arihara K Itoh M. Molecular species-specific differences in composition of triacylglycerols of mouse adipose tissue and diet. Nutr Res. (2008) 28:258–62. 10.1016/j.nutres.2008.02.007 19083417

[38] Marcher AB Loft A Nielsen R Vihervaara T Madsen JGS Sysi-Aho M. RNA-Seq and mass-spectrometry-based lipidomics reveal extensive changes of glycerolipid pathways in brown adipose tissue in response to cold. Cell Rep. (2015) 13:2000–13. 10.1016/j.celrep.2015.10.069 26628366

[39] Heilbronn LK Coster ACF Campbell LV Greenfield JR Lange K Christopher MJ. The effect of short-term overfeeding on serum lipids in healthy humans. Obesity. (2013) 21:649–59. 10.1002/oby.20508 23640727

[40] Mantovani A Dugo C. Ceramides and risk of major adverse cardiovascular events: A meta-analysis of longitudinal studies. J Clin Lipidol. (2020) 14:176–85. 10.1016/j.jacl.2020.01.005 32067904

[41] Lin Z Han F Lu J Guo J Qi C Wang C. Influence of dietary phospholipid on growth performance, body composition, antioxidant capacity and lipid metabolism of Chinese mitten crab, Eriocheir sinensis. Aquaculture. (2020) 516:734653. 10.1016/j.aquaculture.2019.734653

[42] Vahmani P Ponnampalam EN Kraft J Mapiye C Bermingham EN Watkins PJ. Bioactivity and health effects of ruminant meat lipids. Invited Review. Meat Sci. (2020) 165:108114. 10.1016/j.meatsci.2020.108114 32272342

