# Supplementary material for: Lipid analysis of meat from Bactrian camel (Camelus bacterianus), beef, and tails of fat-tailed sheep using UPLC-Q-TOF/MS based lipidomics
Source: Front Nutr. 2023 Mar 2;10:1053116. doi: 10.3389/fnut.2023.1053116 (PMC10017991; doi:10.3389/fnut.2023.1053116)
Supplement: Supplementary file 3 [file Data_Sheet_1.docx]

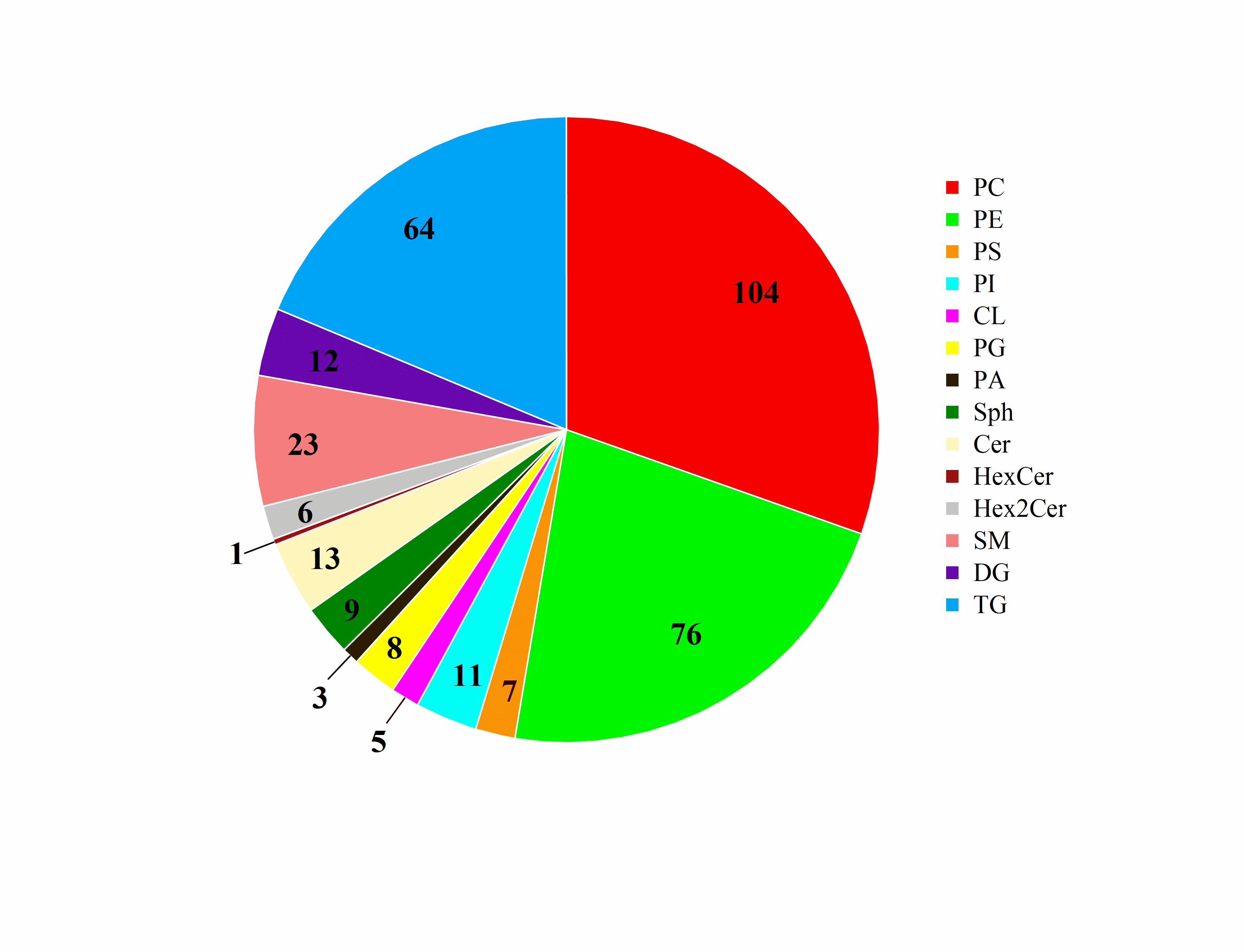


Fig. S1. Lipid composition of four samples (camel meat, camel hump, beef and fatty-tails). PI, phosphatidylinositol; Cer, ceramide; PC, phosphatidylcholine; PE, phosphotidylethanolamine; PS, phosphatidylserine; SM, sphingomyelin; TG, triglyceride; PG, phosphatidylglycerol; Sph, sphinganine; HexCer, hexosylceramide; PA, phosphoric acid; CL, cephalin; DG, diglyceride; Hex2Cer, dihexosylceramide.
